# A Novel *In Silico* Electromechanical Model of Human Ventricular Cardiomyocyte

**DOI:** 10.3389/fphys.2022.906146

**Published:** 2022-06-01

**Authors:** Chiara Bartolucci, Mohamadamin Forouzandehmehr, Stefano Severi, Michelangelo Paci

**Affiliations:** ^1^ Computational Physiopathology Unit, Department of Electrical, Electronic and Information Engineering “Guglielmo Marconi”, University of Bologna, Bologna, Italy; ^2^ BioMediTech, Faculty of Medicine and Health Technology, Tampere University, Tampere, Finland

**Keywords:** computational modeling, human ventricular cardiomyocyte model, action potential (AP), contractility, aftercontraction

## Abstract

Contractility has become one of the main readouts in computational and experimental studies on cardiomyocytes. Following this trend, we propose a novel mathematical model of human ventricular cardiomyocytes electromechanics, BPSLand, by coupling a recent human contractile element to the BPS2020 model of electrophysiology. BPSLand is the result of a hybrid optimization process and it reproduces all the electrophysiology experimental indices captured by its predecessor BPS2020, simultaneously enabling the simulation of realistic human active tension and its potential abnormalities. The transmural heterogeneity in both electrophysiology and contractility departments was simulated consistent with previous computational and *in vitro* studies. Furthermore, our model could capture delayed afterdepolarizations (DADs), early afterdepolarizations (EADs), and contraction abnormalities in terms of aftercontractions triggered by either drug action or special pacing modes. Finally, we further validated the mechanical results of the model against previous experimental and in silico studies, e.g., the contractility dependence on pacing rate. Adding a new level of applicability to the normative models of human cardiomyocytes, BPSLand represents a robust, fully-human in silico model with promising capabilities for translational cardiology.

## 1 Introduction

The future of diagnosis and treatment in cardiology progressively depends on advanced methods in imaging, gene profiling, and pharmaceutical technologies. Despite the recent advances in health technologies, the current empirical clinical investigations face serious challenges as the complexity of therapeutic interventions, prognosis, and the possibility of classifying the treatments grow. Specifically, identifying the optimal treatment strategy with a degree of statistical significance poses serious challenges to current empirical routes in cardiology (Niederer et al., 2019). Furthermore, as precision medicine emerges (Forouzandehmehr et al., 2022), the proven pathophysiological variability between individuals highly augments the detail in the diagnostic process and data, thus, finding an optimal patient-specific solution becomes increasingly difficult (Niederer et al., 2019). Cardiac computational models, based on established physiological and engineering principles, offer a capable framework that not only can be fed by large datasets but also enable mechanistic and integrative simulations leading to disclose novel insights in cardiac pathophysiology (Niederer et al., 2019).

Early physiologically constrained computational models of cardiac cells could quantitatively translate the protein functions into developing cellular phenotypes (Niederer et al., 2019). During the past decade, these models have also incorporated functional characteristics of ion channels, cellular pumps, transporters, and buffers making them promising candidates in preclinical studies (O’Hara et al., 2011; Tomek et al., 2019; Bartolucci et al., 2020; Paci et al., 2021). Currently, as the cardiac contractility data become increasingly available, together with new recording techniques (Ahola et al., 2018), mathematical models of cardiomyocytes (CMS) are developed to predict dynamics of contraction combined with simulation of drug effects alongside the electrophysiology. Toward building models of myocyte electromechanics, elements of contractility have been developed with different levels of complexity focusing on animals (Rice et al., 2008; Campbell et al., 2010; Sheikh et al., 2012; Land et al., 2013) and human CMs (Land et al., 2017). Initial efforts on developing reliable models to capture the electromechanics of human adult CMs have been initiated recently (Lyon et al., 2020; Margara et al., 2021). Margara et al., integrated an established human-based developed contractile element (Land et al., 2017) into the gold standard *in silico* model of human ventricular cell electrophysiology (O’Hara et al., 2011) and into their new ToR-ORd model (Tomek et al., 2019) to predict ventricular active tension generation alongside action potential (AP) and calcium transients (CaT). Also Lyon et al. complemented the ORd model with a contractile element: their choice was the MedChem model of sarcomere mechanics (Dupuis et al., 2018), which they used to assess the impact of β-adrenergic stimulation and sarcomere length on CaTs and force (Lyon et al., 2020).

Our recently published BPS2020 model of the human adult ventricular CM electrophysiology (Bartolucci et al., 2020), holds significant improvements compared with the original ORd model (e.g., the simulation of the experiments with the correct extracellular K^+^ concentration used *in vitro* or the generation of DADs) and includes specific mechanisms not simulated by ToR-ORd (e.g., the inverse APD_90_-[Ca^2+^]_o_ dependence). Given these improvements in simulating electrophysiology phenomena, it is worth investigating how we can expand the spectrum of BPS2020 simulation, by using it as the basis for a new electromechanical human CM *in silico* model.

We have integrated one of the most recent human contractile machinery (LandCE) (Land et al., 2017) into BPS2020 (Bartolucci et al., 2020). As done in Margara et al. (2021), we chose LandCE as it is a contractile element validated against human data. Our goal was to investigate the capabilities of this newly integrated electromechanical model, BPSLand, by evaluating active tension generation and contractility abnormalities e.g., aftercontractions, that can be activated either by drug action or special pacing conditions. BPSLand is a robust, fully-human, *in silico* model meeting the computational expectations from both departments, the electrophysiology and contractility, with the potential for facilitating the translation of biophysical and pharmacological functions into pre-clinical readouts.

## 2 Methods

### 2.1 *In Vitro* Data

To calibrate the BPSLand model, we used a dataset of isometric active tension (Ta) biomarkers recorded from human isolated ventricular CMs (Land et al., 2017; Margara et al., 2021) and a dataset of action potential (AP) biomarkers from human isolated ventricular endocardial CMs (O’Hara et al., 2011; Bartolucci et al., 2020). The Ta biomarker dataset includes measures from strips of the left ventricular myocardium (Mulieri et al., 1992), left ventricular trabeculae (Pieske et al., 1996) and right ventricular trabeculae (Rossman et al., 2004) (additionally considered by Margara et al., 2021). Both datasets were recorded at 37°C. Ta biomarkers are the peak tension (TaPeak), the relaxation time at 50% and 95% (TaRT_50_, TaRT_95_) and the time-to-peak (TaTTP). AP biomarkers are the duration at 30%, 40%, 50%, 70% and 90% (APD_30,_ APD_40_, APD_50_, APD_70_, APD_90_), the maximum upstroke velocity (dV/dt_max_), the peak voltage (VPeak) and the resting membrane potential (RMP). *In silico* biomarkers were computed as in Margara et al. (2021). As we previously reported in Bartolucci et al. (2020), we simulated the AP biomarkers for calibration at [K^+^]_o_ = 4 mM. Conversely, as no information was reported on the *in vitro* Ta biomarker ranges, we run our simulations at the standard concentration [K^+^]_o_ = 5.4 mM.

To validate BPSLand, we used the following human data: 1) APD rate dependence, restitution and accommodation data in control condition and with current blockers from endocardial CMs (O’Hara et al., 2011) (see Supplementary Methods for details and Supplementary Table S1); 2) TaPeak, TaRT_50_ and CaT relaxation time at 50% (CaRT_50_) rate adaptation data (Pieske et al., 1995; Janssen and Periasamy, 2007); 3) TaPeak transmural heterogeneity data from sub-epicardial, mid-myocardial, and sub-endocardial specimens (Haynes et al., 2014).

### 2.2 Integration of the Land Contractile Element Into the BPS2020 Model

The original BPS2020 model (Bartolucci et al., 2020) was based on the seminal O'Hara-Rudy model of the human ventricular AP (O’Hara et al., 2011) and it features two cytosolic compartments, the subspace and the bulk myoplasm, and the sarcoplasmic reticulum (SR) represented with a single compartment. It includes the following ion currents: fast and late Na^+^ currents (I_NaF_, I_NaL_), transient outward K^+^ current (I_to_), L-type Ca^2+^ current (I_CaL_), also with its Na^+^ and K^+^ components (I_CaNa_, I_CaK_), the rapid, slow and inward rectifying K^+^ currents (I_Kr_, I_Ks_, I_K1_), the Na^+^/Ca^2+^ exchanger divided in its cytosolic and subspace components (I_NCXi_, I_NCXss_), the Na^+^/K^+^ pump (I_NaK_), Na^+^, K^+^ and Ca^2+^ background currents (I_Nab_, I_Kb_, I_Cab_) and the sarcolemmal Ca^2+^ pump (I_pCa_). Ca^2+^ fluxes from/to SR are the RyR-sensitive Ca^2+^ release (J_rel_), the SERCA pump (J_up_) and a leakage flux (J_leak_).

We integrated LandCE into BPS2020 following the approach presented in (Margara et al., 2021). Shortly, LandCE takes as input the intracellular Ca^2+^ concentration [Ca^2+^]_i_ computed by BPS2020, to update a new state variable CaTRPN, representing the fraction of troponin C units which bound to Ca^2+^.

$$\frac{dCaTRPN}{dt}=\;k_{TRPN}\left({\left(\frac{{\left[Ca^{2+}\right]}_{i}}{{\left[Ca^{2+}\right]}_{T\text{50}}}\right)}^{n_{TRPN}}\left(\text{1}-CaTRPN\right)-CaTRPN\right)$$

BPS2020 receives as feedback the amount of Ca^2+^ buffered by troponin C, [Ca^2+^]_TRPN_, to update the intracellular Ca^2+^ concentration.

$$\frac{d{\left[Ca^{\text{2}+}\right]}_{i}}{dt}=\;\beta_{Cai}\left(-\left(I_{pCa}+I_{Cab}-\text{2}I_{NaCa,i}\right)\frac{A_{cap}}{\text{2}Fv_{myo}}-J_{up}\;\frac{v_{sr}}{v_{myo}}+\;J_{diff,Ca}\;\frac{v_{ss}}{v_{myo}}-\;\frac{d{\left[EGTA\right]}_{i}}{dt}-\;\frac{d{\left[Ca^{\text{2}+}\right]}_{TRPN}}{dt}\right)$$

$$\frac{d{\left[Ca^{\text{2}+}\right]}_{TRPN}}{dt}={\left[Ca^{\text{2}+}\right]}_{TRPN,max}\frac{dCaTRPN}{dt}$$

where ${\left[Ca^{2+}\right]}_{TRPN,max}$ represents the maximum Ca^2+^ concentration that can bind to troponin C.

### 2.3 Optimization of the BPSLand Model

The structure of the cost function used for both optimizations is the same as in Paci et al. (2018b)

$$Cost=\sum_{1}^{N_{biom}}w_{i}*Cost_{i}$$

$$\mathrm{Cos}t_{i}=\frac{\left(b_{i,\mathrm{sim}}<LB_{i}\right){\left(b_{i,\mathrm{sim}}-LB_{i}\right)}^{2}+\left(b_{i,\mathrm{sim}}>UB_{i}\right){\left(b_{i,\mathrm{sim}}-UB_{i}\right)}^{2}}{0.5\left(LB_{i}+UB_{i}\right)}$$

where *b*_*i,sim*_ is the *i*th simulated biomarker, *LB*_*i*_ the *i*th experimental lower bound for *b*_*i,sim*_, *UB*_*i*_ the *i*th experimental upper bound for *b*_*i,sim*_, *w*_*i*_ the weight for each biomarker’s cost (Supplementary Table S2) and *N*_*biom*_ the number of biomarkers used for optimization. Briefly, if the simulated *i*th biomarker is smaller than *LB*_*i*_ or greater than *UB*_*i*_, the error is computed as the squared distance between the simulated biomarker and the bound, normalized by the center of mass of [*LB*_*i*_, *UB*_*i*_]. Finally, in order to minimize the active tension *T*_*a*_, we included one additional term to Cost, obtaining the final cost function

$${\mathrm{Cost}}_{\mathrm{TOT}}=w_{\mathrm{minTa}}*\min\left(T_{a}\right)+\mathrm{Cost}$$

with $w_{minTa}$ the weight of the minimun active tension.

#### 2.3.1 Step 1: Hybrid Optimization on the LandCE Parameters

After integrating LandCE into BPS2020, we first optimized the LandCE parameters using a hybrid approach combining first a genetic optimization (Matlab function *ga*), followed by the simplex optimization [Matlab function *fminsearchbnd* (D’Errico, 2022)]. The parameters optimized in this first step are only the LandCE parameters listed in Table 1. The optimization ranges for the LandCE parameters are the same as in the original LandCE publication (Land et al., 2017), except for the tropomyosin Ca^2+^ sensitivity ([Ca^2+^]_T50_), for which we chose [0.5, 0.6] instead of [0.8, 0.9]. As the original range [0.8, 0.9] increased substantially the CaT peak, i.e. the systolic Ca^2+^ (+22%), we decided not to affect the BPS2020 electrophysiology and chose [0.5, 0.6] as it preserved the original BPS2020 CaT peak.

**TABLE 1:** Contractility and electrophysiology biomarkers used for the BPSLand optimization, with their ranges.

Model	Step	Parameter	Range
LandCE	1	Tropomyosin rate constant *k*_*u*_ (1/ms)	[0.01, 0.2]
		Hill coefficient *ntm*	[3, 7]
		Unbound-to-weak crossbridge transition scaling factor *ν*	[1, 12]
		Weak-to-strong crossbridge transition scaling factor *μ*	[1, 12]
		Tropomyosin Ca^2+^ sensitivity ([Ca^2+^]_T50_) (μM)	[0.5, 0.6]
BPS2020	2	Maximum Ca^2+^ release flux from SR *J*_*rel,max*_ (1/ms)	[0.016, 0.024]
		Maximum SERCA pump flux *J*_*up,max*_ (mM/ms)	[2.504, 3.756]

For this first optimization step, we considered five contractility and two electrophysiology biomarkers: active tension peak (TaPeak), time-to-peak (TaTTP), relaxation time to 50% and 95% of the diastolic level (TaRT_50_ and TaRT_95_) and the minimum of the diastolic active tension, systolic and diastolic intracellular free Ca^2+^ (CaSys and CaDiast). The acceptable ranges for these biomarkers were taken from the original Land publication (Land et al., 2017) for TaTTP, TaRT_50_ and TaRT_95_, from Margara et al. (2021) for TaPeak, while we chose to set the ranges for CaSys and CaDias as ±5% of their original values (Bartolucci et al., 2020), in order to keep the electrophysiology the most similar to the original BPS2020 model. At the end of this first step, we obtained an electromechanical model whose electrophysiology biomarkers were not significantly affected by the LandCE and correctly simulated TaRT_95_ while the remaining contractility biomarkers were close to their respective lower bounds.

#### 2.3.2 Step 2: Simplex Only

In order to capture the remaining contractility biomarkers, we then run a second simplex optimization on the Ca^2+^ fluxes of the SERCA pump (J_up_) and the RyR-sensitive release (J_rel_), using all the constraints in Table 2, and additional constraints on the AP biomarkers. In particular, for resting potential (RMP), peak voltage (VPeak), maximum upstroke velocity (dV/dt_max_), AP duration at 40%, 50% and 90% (APD_40_, APD_50_ and APD_90_), and the triangulation metric (Tri_9040_), we set the lower and upper bounds as ±5% of their values in the original BPS2020 model, which were fit against the experimental data (O’Hara et al., 2011) at [K^+^]_o_ = 4 mM. We chose these parameters as we did not want to change the ion current parameters of BPS2020, derived from the ORd model and partially fit experimental data in Bartolucci et al. (2020). As ranged for manually tune J_rel,max_ and J_up,max_, we chose ±20% of their original values 20e-3 (1/ms) and 3.13 (mM/ms), respectively. At the end of this second step, the electromechanical model correctly simulated TaRT_95_ and TaPeak while the remaining contractility biomarkers were close to their respective lower bounds. However, we missed one of the key features of BPS2020, i.e., the inverse relationship [Ca^2+^]_o_ – APD_90_, which was otherwise simulated at the end of the first step.

**TABLE 2:** *In vitro* contractility and electrophysiology biomarkers used in the cost function and their goal ranges.

Biomarker	Range [LB, UB]
Active tension peak TaPeak (kPa)	[15, 25]
Active tension time-to-peak TaTTP (ms)	[109, 125]
Active tension relaxation time to 50% TaRT_50_ (ms)	[147, 172]
Active tension relaxation time to 95% TaRT_95_ (ms)	[291, 377]
Minimum active tension min (Ta) (kPa)	—
Systolic intracellular Ca^2+^ CaSys (mM)	[3.004755, 3.321045]e-4
Diastolic intracellular Ca^2+^ CaDias (mM)	[7.712955, 8.524845]e-4

#### 2.3.3 Step 3: Manual Tuning

In order to restore the [Ca^2+^]_o_ – APD_90_ relationship, we added one final step to our pipeline, where we did a minor manual re-tuning of J_rel,max_ (0.0240 →0.0220 1/ms) and J_up,max_ (3.1333 → 3 mM/ms), still considering their lower and upper bounds as in Table 1. The final model is named BPSLand and its parameter values are reported in Table 3. Supplementary Tables S2–S4 summarize the weights, parameters and biomarkers obtained after each of the three optimizations steps. Supplementary Table S5 shows the impact of the manual tuning of J_rel,max_ and J_up,max_ on the [Ca^2+^]_o_ – APD_90_ relationship.

**TABLE 3:** Final BPSLand parameter set.

Parameter	Original value	Optimized value
*ku* (1/ms)	1	1.5230
*Ntm*	5	3.0899
*Ν*	7	1.002
*Μ*	3	2.0779
[Ca^2+^]_T50_ (μM)	0.805	0.5
*J*_*rel,max*_ (1/ms)	20e-3	22e-3
*J*_*up,max*_ (mM/ms)	3.13	3

#### 2.3.4 Rate Dependence

To test the active tension dependence on the applied pacing rate, we paced BPSLand at 0.5, 1, 1.5, 2, 2.5 and 3 Hz for 1,000 beats to reach the steady state, using [K^+^]_o_ = 5.4 mM, [Ca^2+^]_o_ = 1.8 mM and [Na^+^]_o_ = 144 mM as extracellular ion concentration. We then compared simulated TaPeak, TaRT_50_ and CaRT_50_ with the *in vitro* data by Pieske et al., (1995) and Janssen and Periasamy (2007).

#### 2.3.5 Heterogeneity

To simulate transmural heterogeneity, i.e., simulating epicardial (EPI) and mid-myocardial (M) CMs in addition to endocardial (ENDO), we used the same scaling factors reported in Bartolucci et al. (2020) for I_NaL_, I_to_, I_CaL_, I_Kr_, I_Ks_, I_K1_, I_NCX_, I_NaK_, I_Kb_, J_rel_ and J_up_ (Supplementary Table S6).

#### 2.3.6 EAD and DAD Simulations to Trigger Active Tension Abnormalities

We assessed the occurrence of early-afterdepolarization (EADs) and aftercontractions in the BPSLand following three different protocols. First, we simulated the administration of quinidine considering the drug effects on I_Na_, I_Kr_, I_CaL_, I_Ks_ and I_to_, using the IC_50_ and Hill’s coefficients reported in Passini et al. (2017) and Paci et al. (2018a) and the single pore block model, as in Paci et al. (2021) (Supplementary Table S7). We tested three drug concentrations, namely 10, 15 and 20 μM at the standard extracellular ion concentrations ([K^+^]_o_ = 5.4 mM, [Ca^2+^]_o_ = 1.8 mM, [Na^+^]_o_ = 144 mM) and cycle length (CL) of 4,000 ms. The second EAD protocol simulated dofetilide, similarly to what we did in Bartolucci et al. (2020). Shortly, we simulated the administration of 0.1 µM dofetilide at CL = 5,000 ms and extracellular concentrations experimentally used by Guo et al. (2011) ([K^+^]_o_ = 5 mM, [Ca^2+^]_o_ = 2 mM, [Na^+^]_o_ = 137 mM), using the I_Kr_ drug binding values reported by Dutta et al. (2017). We simulated quinidine and dofetilide effects on the endocardial BPSLand model and we anticipate no EADs nor aftercontractions, despite the remarkable AP prolongation. Conversely, the same tests performed on the M cell version, resulted in EADs and aftercontraction.

To assess the occurrence of delayed afterdepolarizations (DADs) we used the same protocol as in Li and Rudy (2011): we fast paced BPSLand for 1,500 beats (BCL = 275 ms) and then we triggered one long beat (BCL = 10,000 ms).

## 3 Results

### 3.1 The BPSLand Model

**FIGURE 1:**
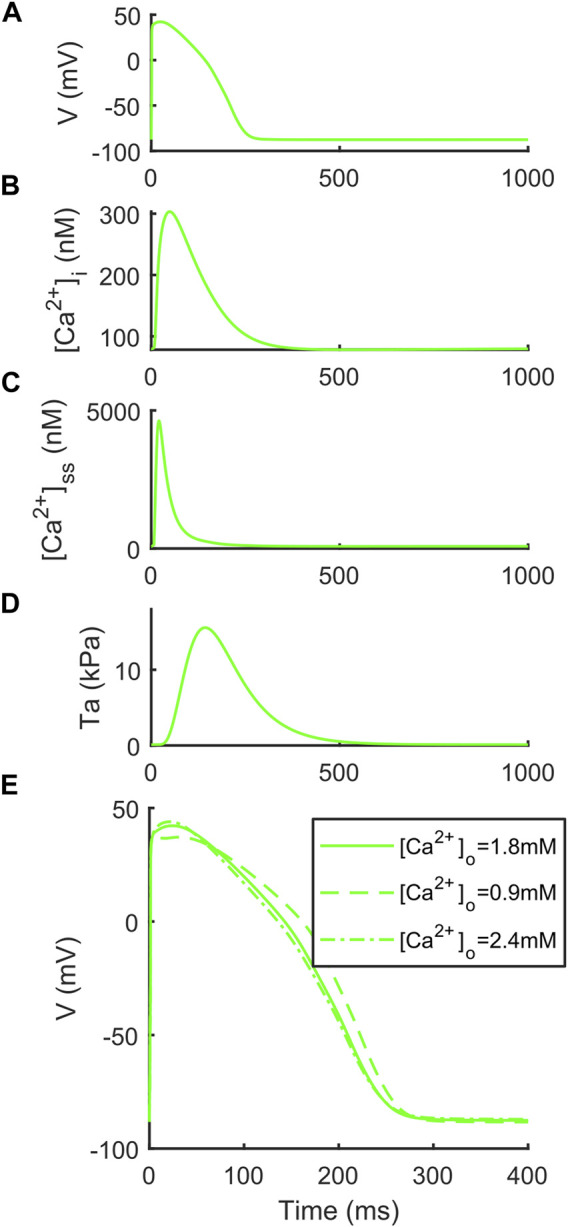
Illustrative traces simulated by BPSLand ([K^+^]_o_ = 5.4 mM). **(A)** Action potential. **(B)** Cytosolic Ca^2+^ concentration. **(C)** Subspace Ca^2+^ concentration. **(D)** Active tension. **(E)** Inverse action potential duration dependence on the extracellular Ca^2+^ concentration.

We report the AP, [Ca^2+^]_i_, [Ca^2+^]_ss_ and Ta traces simulated at [K^+^]_o_ = 5.4 mM in Figure 1, together with the simulations for variable [Ca^2+^]_o_ to highlight the inverse APD_90_-[Ca^2+^]_o_ dependence, which was described first by Severi et al. (2009) and then observed *in vitro* and *in vivo* (Leitch, 1996; Nagy et al., 2013), but failed to be simulated by many *in silico* models, including the original ORd (O’Hara et al., 2011) and ToR-ORd (Tomek et al., 2019). In details, for increasing [Ca^2+^]_o_ = 0.9, 1.8 and 2.4 mM, APD_90_ equals to 251.4, 239.9 and 237.1 ms. Table 4 reports the AP and Ta biomarkers, with the *in vitro* ranges used for the BPSLand calibration, together with additional CaT biomarkers: CaT duration at 50% and 90% (CTD_50_, CTD_90_) and amplitude (CaAmp). All the AP biomarkers are within the experimental ranges, as well as TaPeak and TaRT_95_. Conversely, TaRT_50_ and TaTTP are very close to their respective experimental lower bounds, although out of the *in vitro* ranges. The comparison of AP, Ca^2+^ and Ta traces simulated with [K^+^]_o_ = 4 mM and [K^+^]_o_ = 5.4 mM is presented in Supplementary Figure S1.

**TABLE 4:** The electrophysiology and contractility biomarkers simulated by the original BPS2020 and the new BPSLand models, compared to *in vitro* data.

Biomarker	[K^+^]_o_ = 5.4 mM			[K^+^]_o_ = 4 mM		
	BPS2020	BPSLand	*In vitro*	BPS2020	BPSLand	*In vitro*
APD_90_ (ms)	239.9	239.9	—	267.6	268.4	[180, 440]
APD_50_ (ms)	177.1	175.9	—	200.1	200.0	[110, 350]
APD_40_ (ms)	160.1	158.9	—	178.3	177.3	[85, 320]
Tri_9040_	79.8	81.0	—	89.3	91.1	[50, 150]
dV/dt_max_ (V/s)	248.1	248.8	—	305.3	305.7	[100, 1,000]
VPeak (mV)	42.2	42.2	—	43.7	43.8	[10, 55]
RMP (mV)	-87.6	-87.7	—	-95.6	-95.7	[-103, -88]
CTD_90_ (ms)	247.9	251.3	—	247.6	254.9	—
CTD_50_ (ms)	124.1	138.9	—	125.3	140.3	—
CaSys (nM)	316.3	303.3	—	328.7	311.7	—
CaAmp (nM)	235.1	225.0	—	244.6	230.5	—
CaDias (nM)	81.2	78.2	—	84.1	81.2	—
TaPeak (kPa)	—	15.6	[15, 25]	—	17.4	—
TaTTP (ms)	—	142.9	[147, 172]	—	145.3	—
TaRT_95_ (ms)	—	307.4	[291, 377]	—	308.1	—
TaRT_50_ (ms)	—	108.4	[109, 125]	—	108.2	—
TaMin (kPa)	—	0.100	—	—	0.112	—

### 3.2 Electrophysiology and Contractility Dependence on Pacing Rate

The APD rate adaptation tests reported in Bartolucci et al. (2020) were repeated using BPSLand, to show that introducing LandCE did not affect the capability of the new model in simulating the old data. Briefly, BPSLand simulated the *in vitro* data as satisfactorily as BPS 2020, outperforming the original ORd model (Supplementary Figures S2, S3).

**FIGURE 2:**
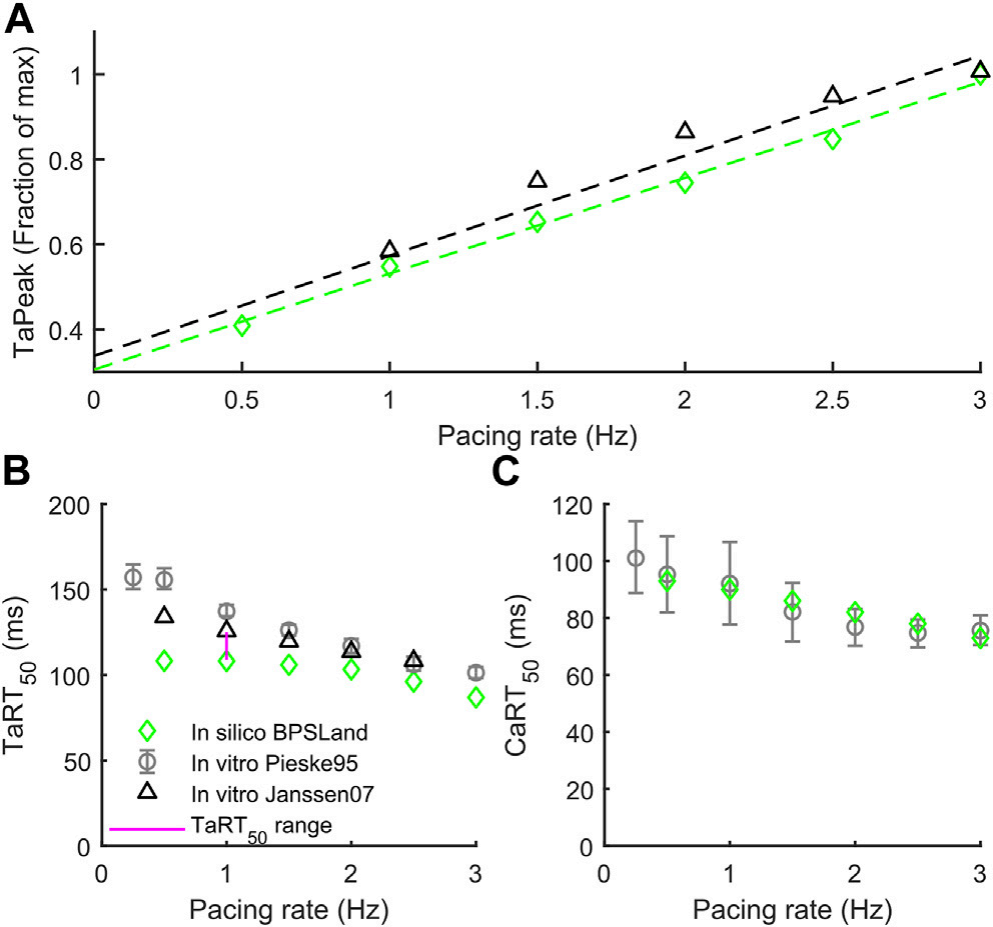
Active tension dependence on pacing rate and comparison with the *in vitro* data from Pieske et al. (1995) and Janssen and Periasamy (2007). **(A)** Normalized active tension peak. **(B)** Active tension relaxation time. **(C)** Ca^2+^ transient relaxation time.

In this section we validate BPSLand against two additional *in vitro* datasets of rate adaptation of TaPeak, TaRT_50_ and CaRT_50_, not considered for BPS2020. Figure 2A shows the qualitative agreement of our model with the data published by Janssen and Periasamy (2007) in terms of TaPeak-pacing rate dependence. In particular, we successfully simulate the linearity of such dependence. In Figure 2B, we considered the rate dependence of TaRT_50_, considering *in vitro* data by Pieske et al., 1995 and Janssen and Periasamy (2007). BPSLand simulations qualitatively agree both with the Janssen07 and the Pieske95 experiments, although TaRT_50_ is lower at the slowest pacing rates. This discrepancy is due to the TaRT_50_ *in vitro* range used to calibrate the BPSLand model at 1 Hz, i.e. [109, 125] ms (purple line). BPSLand is positioned at the interval lower bound (108.4 ms), while Janssen07 data at the upper bound (125 ms) and Pieske95 is out of bound (137.2 ms). Conversely, BPSLand shows quantitative agreement with the Pieske95 CaRT_50_ data (Figure 2C). A comparison with the ToR-ORd+Land model is shown in Supplementary Figure S4. Furthermore, the length dependence properties of the BPSLand model is presented in Supplementary Figure S5 and was performed in the same way proposed by Margara et al. (2021) in their original Supplementary Section S6.

### 3.3 Transmural Heterogeneity

**FIGURE 3:**
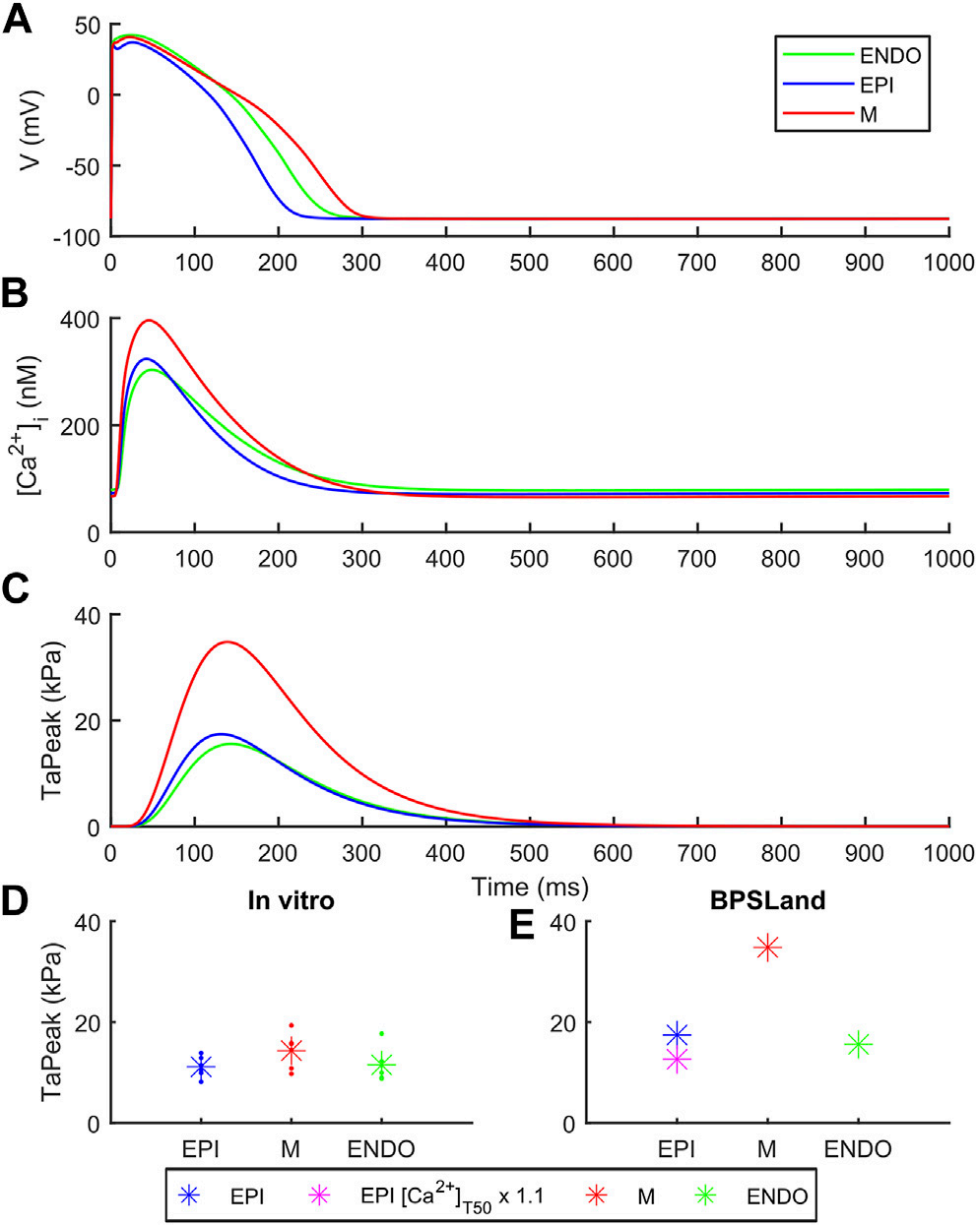
Transmural heterogeneity simulations with BPSLand in endocardial (green), epicardial (blue) and mid-myocardial (red) modes. **(A)** Action potentials. **(B)** Cytosolic Ca^2+^ concentration. **(C)** Active tension. **(D–E)** Comparison of the simulated and experimental active tension peak magnitude across endocardial, epicardial and mid-myocardial cell types. The simulated purple star represents an additional epicardial simulation where we tested a small increments of the calcium sensitivity, ×1.1 the baseline [Ca^2+^]_T50_ value, to obtain an active tension even closer to the experimental data (Haynes et al., 2014).

Figure 3 shows how BPSLand simulates the transmural heterogeneity in terms of electrophysiology and contractility. Our simulations are in agreement with the ToR-ORd+Land and ORd+Land models presented in Margara et al. (2021). In terms of APD, the M model has the longest APs, followed by the ENDO and EPI models. In terms of CaTs and active tension, the M model shows the highest peaks, followed by EPI and ENDO. Haynes et al. reported transmural heterogeneity data of isometric active tension peaks in human heart preparations, showing similar average active tension in EPI and ENDO preparations (although EPI < ENDO), and greater in M specimens (Haynes et al., 2014). We simulate an EPI TaPeak (17.4 kPa) slightly greater than ENDO (15.6 kPa), while the M model produces greater TaPeak (34.8 kPa). This is the same trend simulated by the ToR-ORd+Land model (TaPeak M > EPI > ENDO), although the absolute TaPeak values are considerably greater in ToR-ORd+Land than in BPSLand. As in (Margara et al., 2021) the authors suggested that the Ca^2+^ sensitivity in ENDO CMs could be higher than in EPI cells, we tested how much upscaling of [Ca^2+^]_T50_ is required in EPI BPSLand to bring the simulated EPI TaPeak even closer to the experiments (Haynes et al., 2014). In fact, [Ca^2+^]_T50_ is not considered as one of the parameters to change when switching from ENDO to EPI models. The purple star in Figure 3E show that a ×1.1 upscale produces an EPI TaPeak matching the experiments. A comparison of the transmural heterogeneity with the ToR-ORd+Land model is also reported in Supplementary Figure S6.

### 3.4 EADs, DADs and Aftercontractions

The endocardial BPSLand model did not produce EADs just by administering quinidine or dofetilide, despite the extreme APD_90_ prolongation up to +272% with 0.1 µM dofetilide; +398%, +489%, +563% with the three increasing quinidine doses.

**FIGURE 4:**
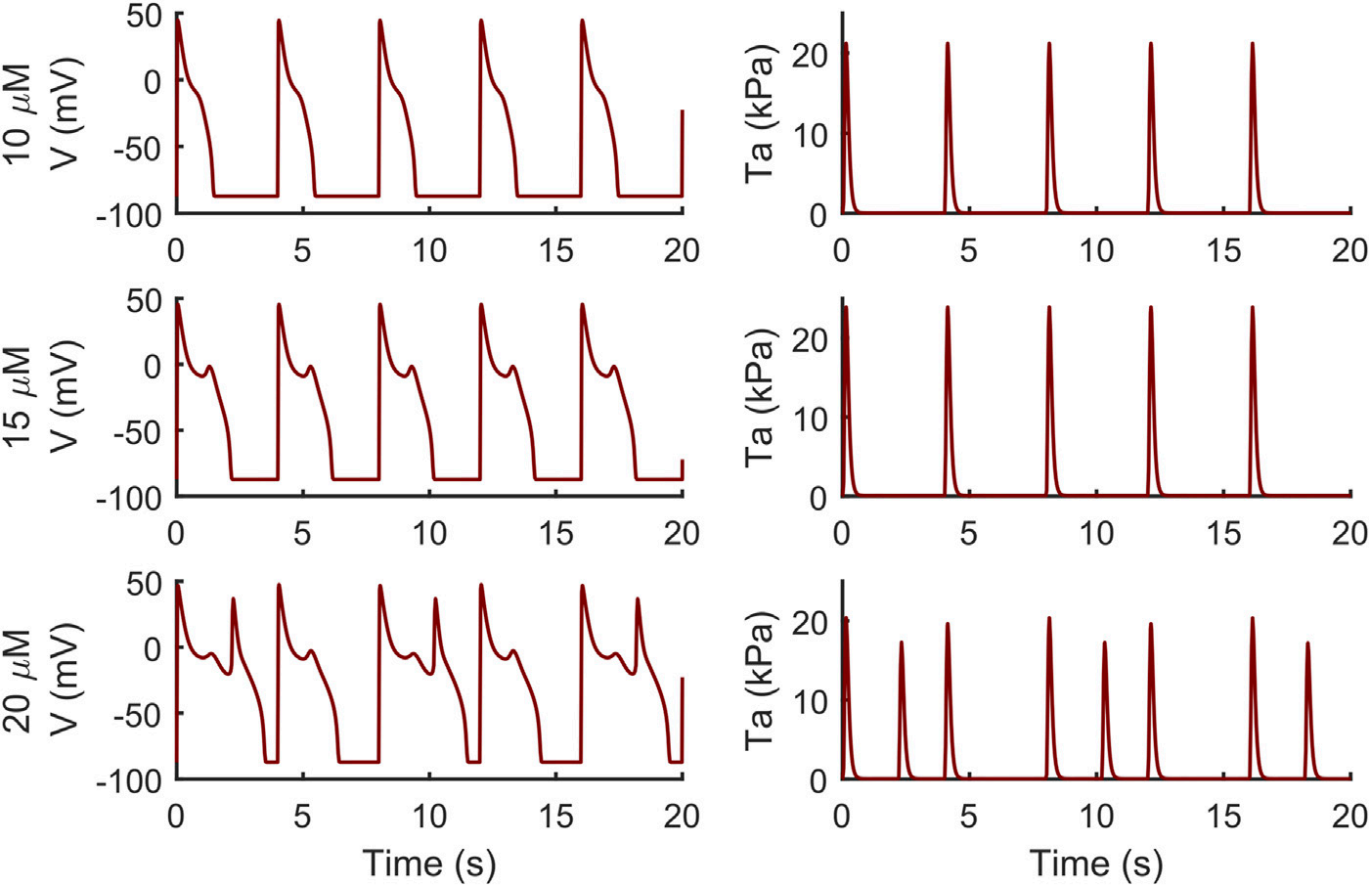
Illustrative traces of the membrane potential (left column) and active tension (right column) simulated by the M cell BPSLand with low (10 µM), intermediate (15 µM) and high (20 µM) quinidine concentrations. The intermediate and high doses trigger early afterdepolarizations and aftercontractions.

**FIGURE 5:**
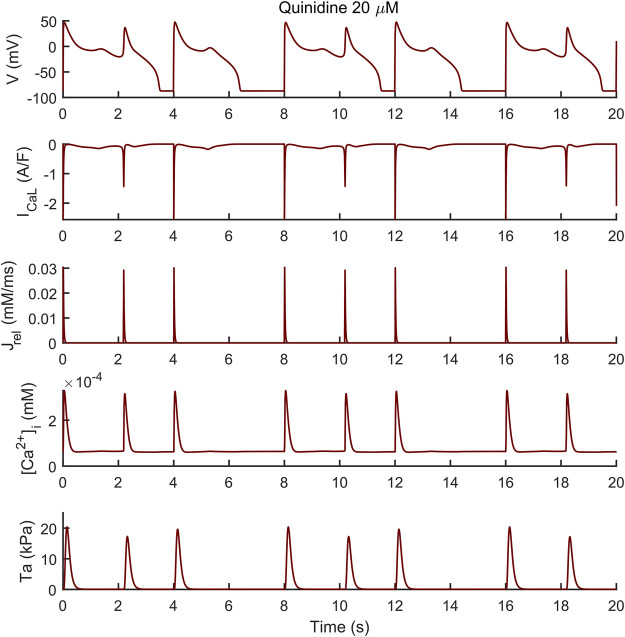
Early afterdepolarizations (EADs) triggered by 20 µM of quinidine and their underlying mechanisms in the M cell BPSLand. The smaller EADs due to I_CaL_ reactivation (I_CaL_-driven), e.g., *t* ∼ 1.3 s or t ∼ 5.3 s do not have a corresponding aftercontraction. Conversely, EADs trigger by a spontaneous Ca^2+^ release from the sarcoplasmic reticulum through J_rel_, e.g., *t* = ∼ 2.2 s or *t* = ∼ 10.2 s, have a corresponding aftercontraction, since J_rel_ pours into the cytosol enough Ca^2+^ to trigger the contractile element.

Conversely, the M cell version, characterized by smaller I_Kr_ and larger I_CaL_, reacted to both drugs with EADs and, in some cases, aftercontractions. The simulations shown in Figure 4 are noteworthy: for both the intermediate and high quinidine concentrations (15 µM in the second row and 20 µM in the third row) quinidine triggers EADs, but only some of them have a correspondent aftercontraction. This is due to the different mechanisms underlying each EAD and it is well summarized in case of 20 µM quinidine, reported in more detail in Figure 5. The smaller EADs due to I_CaL_ reactivation (I_CaL_-driven), e.g., *t* ∼ 1.3 s or *t* ∼ 5.3 s do not have a corresponding aftercontractions. On the other hand, other EADs are triggered by a spontaneous Ca^2+^ release from the SR through J_rel_, e.g., t ∼ 2.2 s or t ∼ 10.2 s, which pours into the cytosol enough Ca^2+^ to trigger the contractile element to produce an aftercontraction. Therefore, from this result, we can hypothesize there is not a 1:1 EAD-aftercontraction correspondence, since aftercontractions require enough Ca^2+^ to start, as in the case of J_rel_ intervention.

**FIGURE 6:**
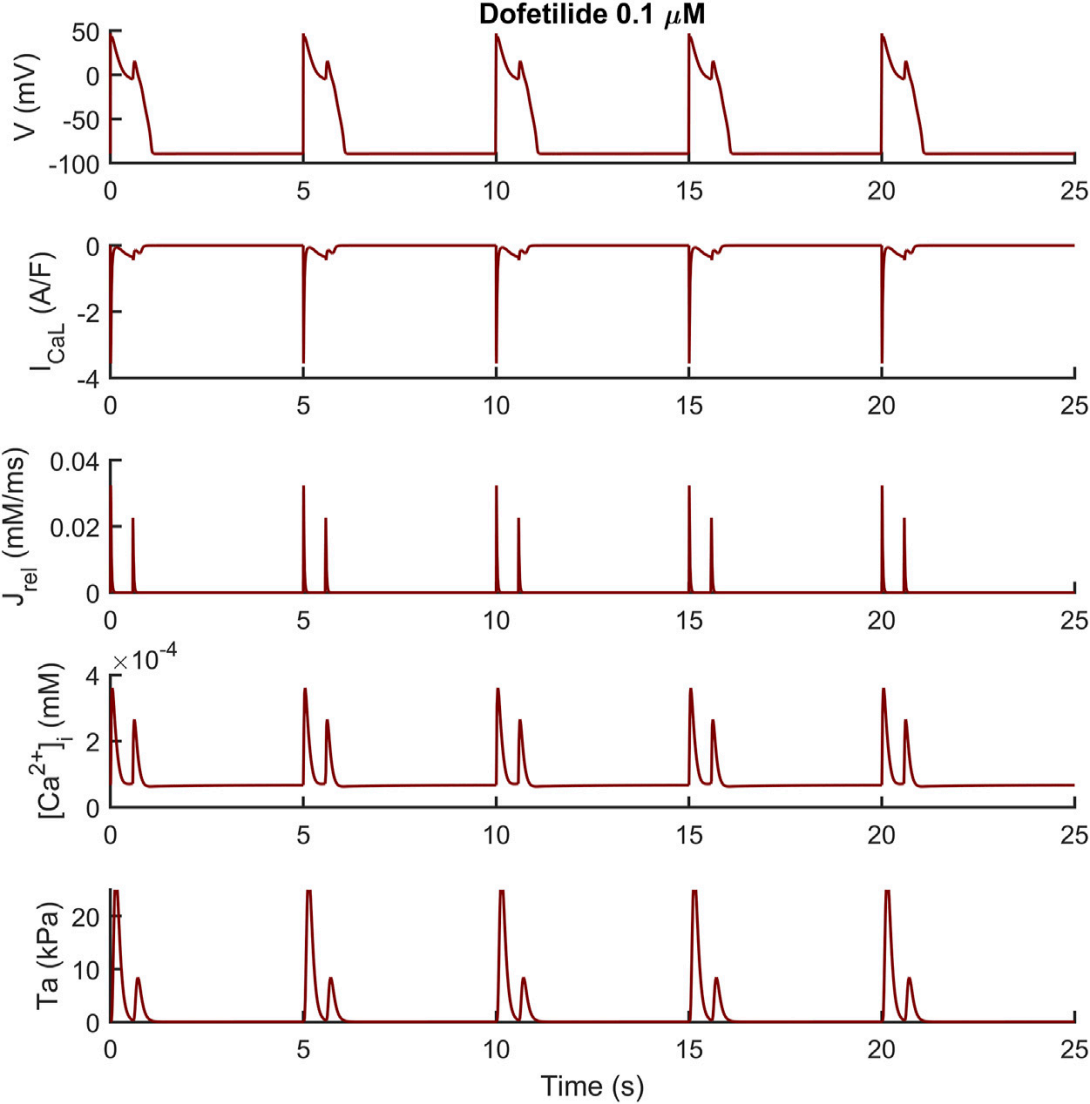
Illustrative early afterdepolarizations and aftercontractions triggered by 0.1 µM of dofetilide in the M cell BPSLand.

We observed a similar result with dofetilide in Figure 6 where the dofetilide simulation resulted in EADs and aftercontractions. Also in this case, the EADs are triggered by spontaneous Ca^2+^ release from the SR through J_rel_, as shown in the third panel.

**FIGURE 7:**
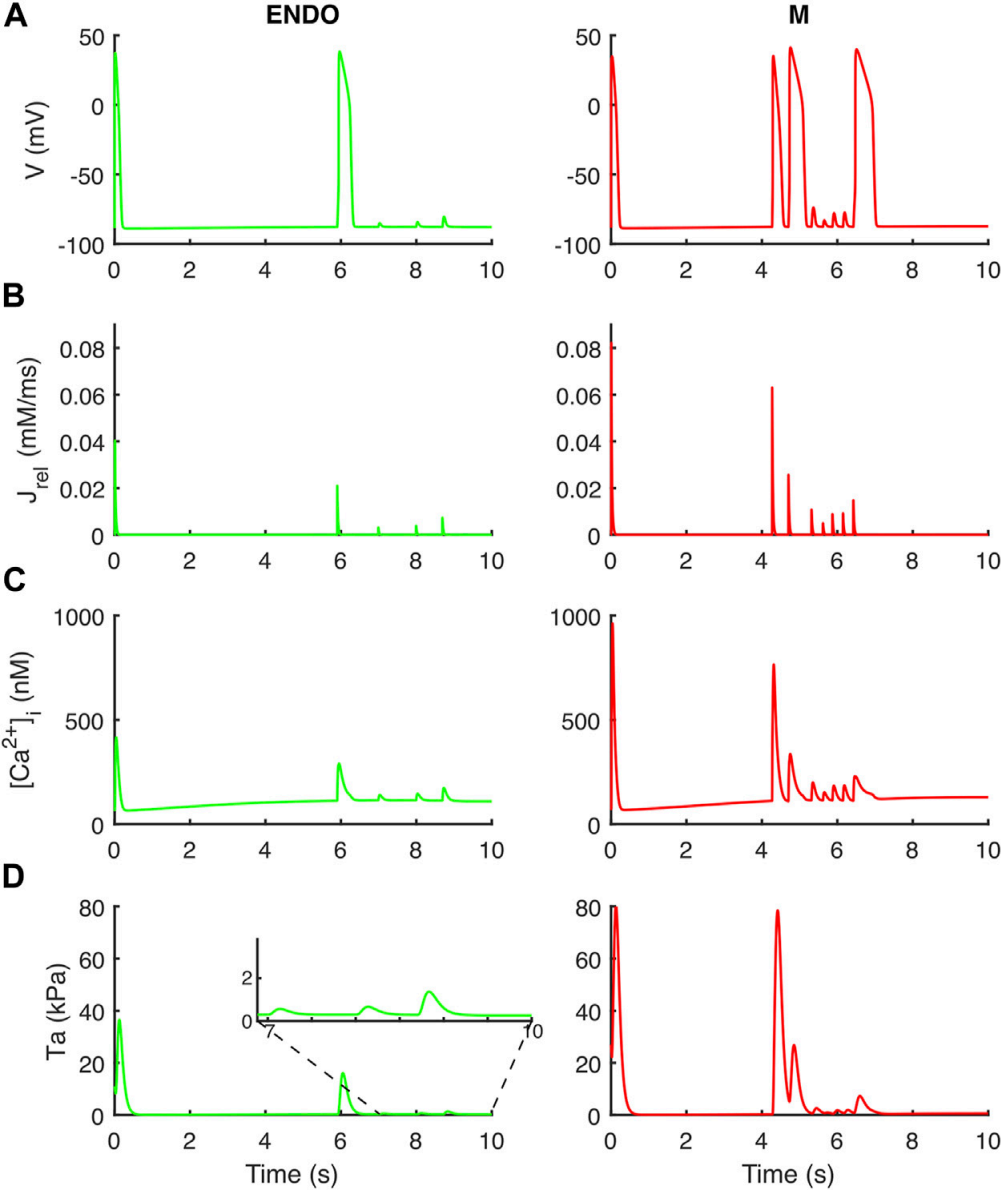
Aftercontractions triggered by anticipated beats and DADs in the endocardial (left column) and M cell (right column) BPS 2020. The action potential at *t* = 0 is the long beat at BCL = 10,000 ms, following 1,500 beats at BCL = 275 ms. The action potential at t ∼ 6 s (left) and *t* ∼ 4.2 s/4.7 s/6.4 s (right) is triggered by the spontaneous Ca^2+^ release from the sarcoplasmic reticulum and not by external pacing. **(A)** Membrane potential. **(B)** Ca^2+^ release flux from the sarcoplasmic reticulum. **(C)** Cytosolic Ca^2+^ concentration. **(D)** Active tension with aftercontractions. The zoomed inset on the left column highlights the small aftercontractions corresponding to the DADs following the anticipated action potential.

Following the DAD Li et al. protocol, BPSLand triggered an unpaced beat further followed by several DADs (Figure 7). The fast pacing protocol led to the accumulation of Ca^2+^ in the SR (oscillations in [1.76, 2.12] mM instead of [1.20, 1.47] mM), which was spontaneously released by J_rel_ during the diastolic phase of the last long beat. These unpaced releases of sarcoplasmic Ca^2+^ not only triggered the anticipated AP and DADs (as we already showed in Bartolucci et al., 2020), but it also was enough to trigger aftercontractions (Figure 7D inset).

## 4 Discussion

In this work, we present an updated version of our BPS2020 model of the human ventricular AP (Bartolucci et al., 2020), that we enhanced with the contractility model presented by Land et al. (2017). The potential of *in silico* models is getting more and more recognition both by industry and regulators for specific applications, e.g., cardiac safety pharmacology (Li et al., 2020; Musuamba et al., 2021). However, most of the current cardiac cell models focus mainly on electrophysiology, i.e., AP and Ca^2+^ handling, not considering the fact that the heart behaves like a pump, and therefore the contractile activity of CMs is surely worth of interest. Most of the diseases of interest modelled so far within silico CM models mainly affected specific ion channels [long QT syndrome (Clancy and Rudy, 2002; Paci et al., 2017, 2018a; Kernik et al., 2020)] or Ca^2+^ handling [catecholaminergic polymorphic ventricular tachycardia (CPVT) (Koivumäki et al., 2018)]. Conversely, hypertrophic cardiomyopathy (HCM), the most widespread genetic cardiac disorder, primarily associates with pathogenic variants in protein genes of sarcomere (Santini et al., 2020). In fact, most of pathogenic variants in HCM are hosted by myosin binding protein C and adult cardiac myosin isoforms that are mainly programmed by MYBPS3 and MYH7 genes, respectively, (Toepfer et al., 2020). These variants are responsible for myocardium hypercontractility (Sarkar et al., 2020), impaired contractile relaxation (Toepfer et al., 2020), arrhythmogenesis, diastolic dysfunction and heart failure (Sarkar et al., 2020). Furthermore, the hypoxia-induced lack of oxygenation in ischemia impairs the orchestrate of molecular events leading to normal ventricular contraction (Katz, 1973). Finally, the glycation of myofilaments in diabetes, a major risk factor in heart failure, correlates with significant reduction in calcium sensitivity of the sarcomere (Papadaki et al., 2022) that cannot be captured in electrophysiology-only models. The same applies to new drugs directly targeting sarcomere dynamics, e.g., blebbistatin, omecamtiv mecarbil and mavacamten (Rahman et al., 2018; Awinda et al., 2020; Fülöp et al., 2021): with no *in silico* contractility description, it is not possible to properly simulate them.

Therefore, the goal of our work was to provide the new and validated BPSLand *in silico* model of human adult CMs, combining both electrophysiology and contractility. As the electrophysiology description by BPS2020 carried a few novelties, especially the APD-[Ca^2+^]_o_ relationship and an extended and more reliable description of Ca^2+^ handling, including the generation of DADs, it was important to us to create a model able to translate such novelties also to contractility. Of note, we did not aim to simulate here specific pathological conditions affecting contractility, as this will be topic for future works.

### 4.1 Development of the BPSLand Model

We followed the same strategy published by Margara et al. (2021) for their ToR-ORd+Land model, to integrate the electrophysiology described by BPS2020 and the contractility of LandCE: as forward mechanism, LandCE takes as input the cytosolic Ca^2+^ concentration computed by BPS2020, to compute the fraction of troponin C units bound to Ca^2+^, and this new flux of Ca^2+^ towards the sarcomere is then included in the equation regulating the BPS2020 cytosolic Ca^2+^ concentration, to close the loop. In terms of mathematical formulation, the process was straightforward, as BPS2020 and ToR-ORd are both based on the original ORd model. For the optimization of the model, we built our cost function with the same biomarkers (TaPeak, TaTTP, TaRT_50_ and TaRT_95_) and experimental ranges as in Land et al. and Margara et al., and we tuned the same parameters (k_u_, ntm, ν, µ and [Ca^2+^]_T50_) within the same ranges, except for the Ca^2+^ sensitivity [Ca^2+^]_T50_. In both Land et al. and Margara et al., [Ca^2+^]_T50_ was optimized within [0.8, 0.9]. However, values in that range would have altered too much the CaT amplitude of BPS 2020. Land et al. already reported that such parameter “needs to be fit depending on the calcium transient used to drive the model,” as it is not consistent inter-species and also variable in their experiments on skinned human CMs. Therefore, we optimized [Ca^2+^]_T50_ in the range [0.5, 0.6], which allowed us to keep the same CaT morphology and magnitude of the original BPS 2020. As we reported in Section 2, we followed a hybrid optimization approach based on genetic algorithm (Step 1, as in Land et al. and Margara et al.) to avoid being stuck in local minima, followed by a simplex (Step 2) on the sarcoplasmic Ca^2+^ fluxes again to keep the BPSLand Ca^2+^ handling the most similar to BPS2020. Already at this stage, the resulting model would have satisfactorily simulated the considered AP and Ta biomarkers. However, it lost the ability to simulate the inverse APD-[Ca^2+^]_o_ dependence for high [Ca^2+^]_o_ values. Such dependence was one of the key-novelties of BPS2020 (Bartolucci et al., 2020). In order to restore it (Figure 1), we added one step of manual tuning on the sarcoplasmic maximal fluxes J_rel,max_ and J_up,max_, applying only minor changes fully consistent with the physiological formulation (Step 3). The final BPSLand model satisfactorily simulates AP and Ta biomarkers, together with the APD-[Ca^2+^]_o_ inverse dependence (Figure 1; Table 4).

### 4.2 Validation of the Model Against *In Vitro* Data and Comparison With Other *In Silico* Models

We first validated BPSLand against the same AP data (APD rate dependence and restitution in control condition and with current blocker) used to validate BPS2020 and the original ORd model. The rationale is we want BPSLand to work as well as BPS2020 in simulating electrophysiology data. As we already presented in detail those simulations in Bartolucci et al. (2020), here we report our results and the used protocols in the Supplementary Section S1 and Supplementary Figures S2, S3). These results confirm that adding the mechanical model have not altered the behaviour of the model electrophysiology. Nevertheless, it should be taken into account that having a single experimental dataset, including both electrical and mechanical measurements, would be the ideal setting to better calibrate and validate an electromechanical model (same *in vitro* preparations, clearer assessment of the mechanoelectric feedback, etc.). However, to our current knowledge, there is no such kind of data collection.

In terms of contractile properties, we compared BPSLand simulations to *in vitro* experiments performed on human CMs and cardiac preparations (Section 2.1). BPSLand successfully simulated the linear force-frequency dependence reported by Janssen and Periasamy (2007) (Figure 2). Such dependence was previously simulated by Lyon et al. (2020), although obtaining lower values of normalized force compared to BPSLand and to *in vitro* data in the range [1, 2.5] Hz (see Figure 2B in the original Lyon et al. paper). In terms of relaxation time, BPSLand optimally replicated the CaRT_50_ data by Pieske et al., and very well the TaRT_50_ by Pieske et al. (1995) and Janssen and Periasamy (2007). BPSLand TaRT_50_ is lower at the slowest pacing rates (Figure 2). We ascribe this discrepancy to the TaRT_50_ interval we used at 1 Hz during the model optimization: BPSLand and Janssen07 TaRT_50_ are positioned at the opposite sides of such interval (purple line in Figure 2) while Pieske95 is out of bound. In terms of transmural heterogeneity (Figure 3), BPSLand simulations are in agreement with the *in silico* results of ToR-ORd+Land in terms of APD (M > ENDO > EPI) and TaPeak (M > EPI > ENDO) sequences, although the TaPeak values are greater in ToR-ORd+Land than in our model (M ∼ 60 kPa, EPI ∼ 40 kPa, ENDO ∼ 20 kPa). In fact, although we used the same TaPeak range as in Margara et al. (2021), i.e., [15, 25] kPa, BPSLand simulates a reference ENDO TaPeak equals to 15.6 kPa, which is more in line with the Haynes et al. (2014) *in vitro* values (Figure 3), especially for the small difference we observed in our ENDO vs. EPI TaPeak. As in Margara et al. (2021), we have tested heterogeneities in myofilament calcium sensitivities by acting on the baseline [Ca^2+^]_T50_ value for the epicardial cell type (Figure 3E), showing that a small change of the [Ca^2+^]_T50_ parameter replicates better the experiments. This result suggests that simulating transmural heterogeneity with electromechanical models may not only require re-calibration of the electrophysiological part but also of the mechanical part of the chosen model (Haynes et al., 2014).

Abnormalities in the ionic regulations of cardiomyocytes e.g., EADs and DADs can trigger the occurrence of a contractile irregularity in form of aftercontractions (Nguyen et al., 2017) the incidence of which has been reported in animal models of heart failure associated with arrhythmogenesis (Pogwizd et al., 2001). We observed that the endocardial BPSLand, as the original BPS2020, reacts to dofetilide and quinidine not producing EADs nor aftercontractions, but with an extreme prolongation of APD. This is not surprising, since we designed BPSLand carefully maintaining the electrophysiology of BPS2020. On the other hand, the M cell model reacted to both drugs with such abnormalities in electrophysiology and contractility. From the modelling point of view, it is not surprising: compared to the endocardial model, the M cell model has smaller G_Kr_ (thus smaller repolarization reserve), larger G_CaL_ (thus being prone to more significant reactivation of I_CaL_ during phase 3 of the AP) and J_rel,max_ (i.e., larger releases, also spontaneous, of Ca^2+^ from SR). From the *in vitro* point of view, the higher sensitivity of M cells to drugs affecting repolarizing ion currents compared to endocardial and epicardial was reported by Antzelevitch et al. (1999), with a panel of 13 drugs. Nonetheless, we did not observe a 1:1 correspondence between EADs and aftercontractions. We previously observed (see Figure 6C in the original BPS2020 paper) EADs triggered by two different mechanisms: I_CaL_ reactivation-driven and RyR spontaneous opening-driven EADs, as we also report here in Figure 5. Only in the case of a RyR spontaneous opening-driven EAD, we also have the corresponding aftercontraction, which is not present for I_CaL_-driven EADs. Similarly to RyR spontaneous opening-driven EADs, also DADs are source of aftercontractions (Desantiago et al., 2008). BPSLand correctly simulated them using a protocol aimed to stress the model. Aftercontractions have been reported *in vitro* following the administration of diverse compounds or in presence of mutations in several cardiac preparations, e.g., cardiac tissues and trabeculae following dofetilide administration (Nguyen et al., 2017), in myocardial slices containing titin and collagen administered with isoproterenol (Watson et al., 2019), or in CPVT human induced pluripotent stem cell-derived CMs (Novak et al., 2012).

### 4.3 Limitations

The proposed computational model can be used to better understand the electromechanical interactions and the strong relationship between Ca^2+^ regulation and mechanics. Despite this, the experimental *in vitro* human data, taking into account both electrical and mechanical aspects, are still few, and urgently required to ensure better insight in electromechanical coupling and design more accurate models. The BPSLand model itself has some limitations. Preload and afterload conditions contribute to contractility response and should be considered in future model developments by including a mathematical description of dynamic changes in sarcomere length, since now only the isometric condition can be simulated. Other previously published mechanical models, e.g., Rice et al. (2008), Dupuis et al. (2016), and Dupuis et al. (2018), also include a mechanical description of sarcomere lengthening and shortening, thus expanding the range of possible simulations. Our choice to use Land model is based on the fact that it is validated against human experimental data. BPSLand model describes mechano-electric feedback only through the binding of calcium to troponin, but this phenomenon also includes other actors, for example stretch-activated ion channels (Peyronnet et al., 2016), which are modulated by membrane stretch and yield a current acting on the cardiomyocyte membrane potential. Future works should include into the model also these channels. Figures 2B,C show another limitation of BPSLand: while the model captures well the CaRT_50_ *in vitro* data, it slightly underestimates the TaRT_50_, as BPSLand simulates very similar CaRT_50_ and TaRT_50_ for each tested rate. One reason could be a slightly too fast relaxation dynamic in the contractile element. However, we replicated the same test with a second *in silico* model (Supplementary Figure S4) and an even more different behavior emerges. As cardiomyocyte electromechanical models are not so common yet as electrophysiology only models, it is clear that further iterations of optimization and validation shall be made in the future. Finally, we did not test the application of the model to multiscale simulations (2D or 3D) since it was beyond the scope of the work, although it will be interesting to check BPSLand behaviour also in this field of applicability.

## 5 Conclusion

In this paper, we presented our new electromechanical model of human adult ventricular cardiomyocyte, built and validated using several sets of human *in vitro* experiments. In addition to replicate correctly the results produced by its predecessor BPS2020, BPSLand adds an accurate simulation of active tension and contractility abnormalities, which can be triggered by drugs or specific pacing protocols. Therefore, BPSLand expands the domain of applicability of *in silico* model, which traditionally focus mainly on the simulation of the cardiac cell electrophysiology.

## Data Availability

The original contributions presented in the study are included in the article/Supplementary Material, further inquiries can be directed to the corresponding author.
