# Supplementary material for: A Novel In Silico Electromechanical Model of Human Ventricular Cardiomyocyte
Source: Front Physiol. 2022 Jun 1;13:906146. doi: 10.3389/fphys.2022.906146 (PMC9198403; doi:10.3389/fphys.2022.906146)
Supplement: Supplementary file 1 [file DataSheet1.PDF]

Supplementary Material

A novel in silico electromechanical model of human ventricular cardiomyocyte

Chiara Bartolucci[*], Mohamadamin Forouzandehmehr, Stefano Severi, Michelangelo Paci

*** Correspondence:** Chiara Bartolucci: chiara.bartolucci4@unibo.it

1 Supplementary Methods

1.1 APD Rate Dependence

APD rate dependence (Figure S2A, left panel) was computed at steady state for different CLs, while APD restitution (Figure S2A, right panel) was computed using the S1/S2 protocol, i.e., steady state at CL = 1,000 ms (S1), followed by a single S2 extra-systolic stimulus, delivered at various diastolic intervals (DIs). APDs at 30, 50, 70, and 90% of repolarization were compared against the human experimental data from (O'Hara et al., 2011). APD rate dependence and restitution were tested in presence of specific channel blockers (Figure S2B-C). Current block percentages were refined as in (Bartolucci et al., 2020) (Table S1). Simulations for all these protocols were run with the following extracellular ion concentrations: $[K^+]_o$ = 4 mM, $[Ca^{2+}]_o$ = 1.8 mM and $[Na^+]_o$ = 144 mM as in the experiments from (O'Hara et al., 2011).

Table S1: % of specific channel blockers used in the simulations for APD rate dependence and restitution.

Simulated Drugs	Affected current	Block percentage	Reference
1mM E-4031	I_{Kr}	70%	(O'Hara et al., 2011)
1mM HMR-1556	I_{Ks}	90%	(O'Hara et al., 2011)
1mM nisoldipine	I_{CaL}	100%	(Walsh et al., 2007)
100mM $BaCl_2$	I_{K1}	84%	(Biliczki et al., 2002)
1mM mexiletine	I_{NaL}, I_{CaL}, I_{Kr}	54%, 20%, 9%	(Dutta et al., 2017)
5mM ryanodine	J_{rel}	30%	(Zucchi and Ronca-Testoni, 1997; Tripathy et al., 1998; Thomas and Williams, 2012)

1.2 APD Accommodation

APD accommodation, i.e., the time course of APD response to abrupt changes in pacing rate, was measured in human patients by (Franz et al., 1988): the same protocol was simulated with both BPS2020 and BPSLand model (Figure S2B) and compared with the experimental data (Figure S2A).

2 Supplementary Figures

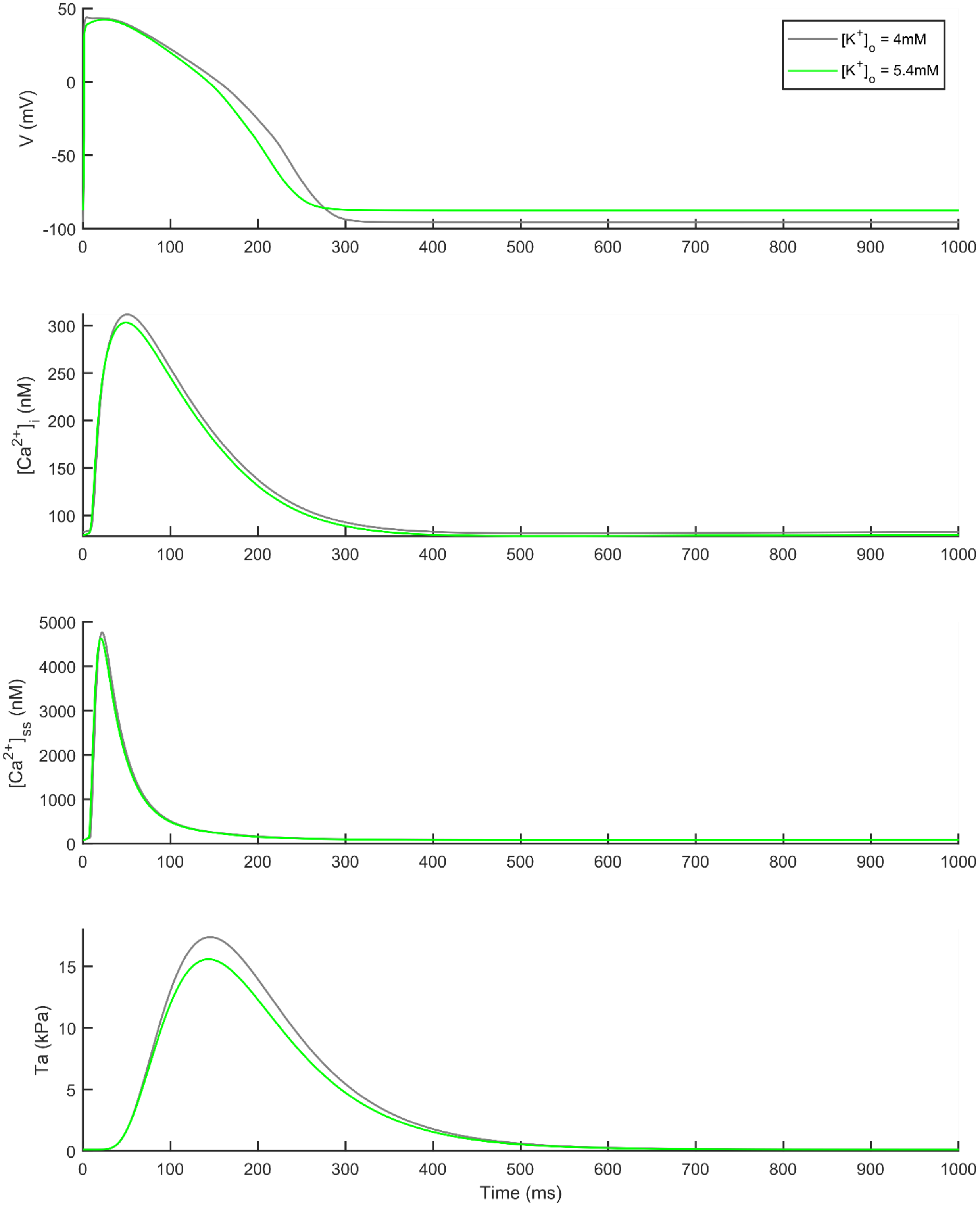

Figure S1. Illustrative comparison of action potential, cytosolic Ca^{2+} concentration, subspace Ca^{2+} concentration, and active tension, simulated by BPSLand at $[K^+]_o$ = 4 mM (grey) vs $[K^+]_o$ = 5.4 mM (green).

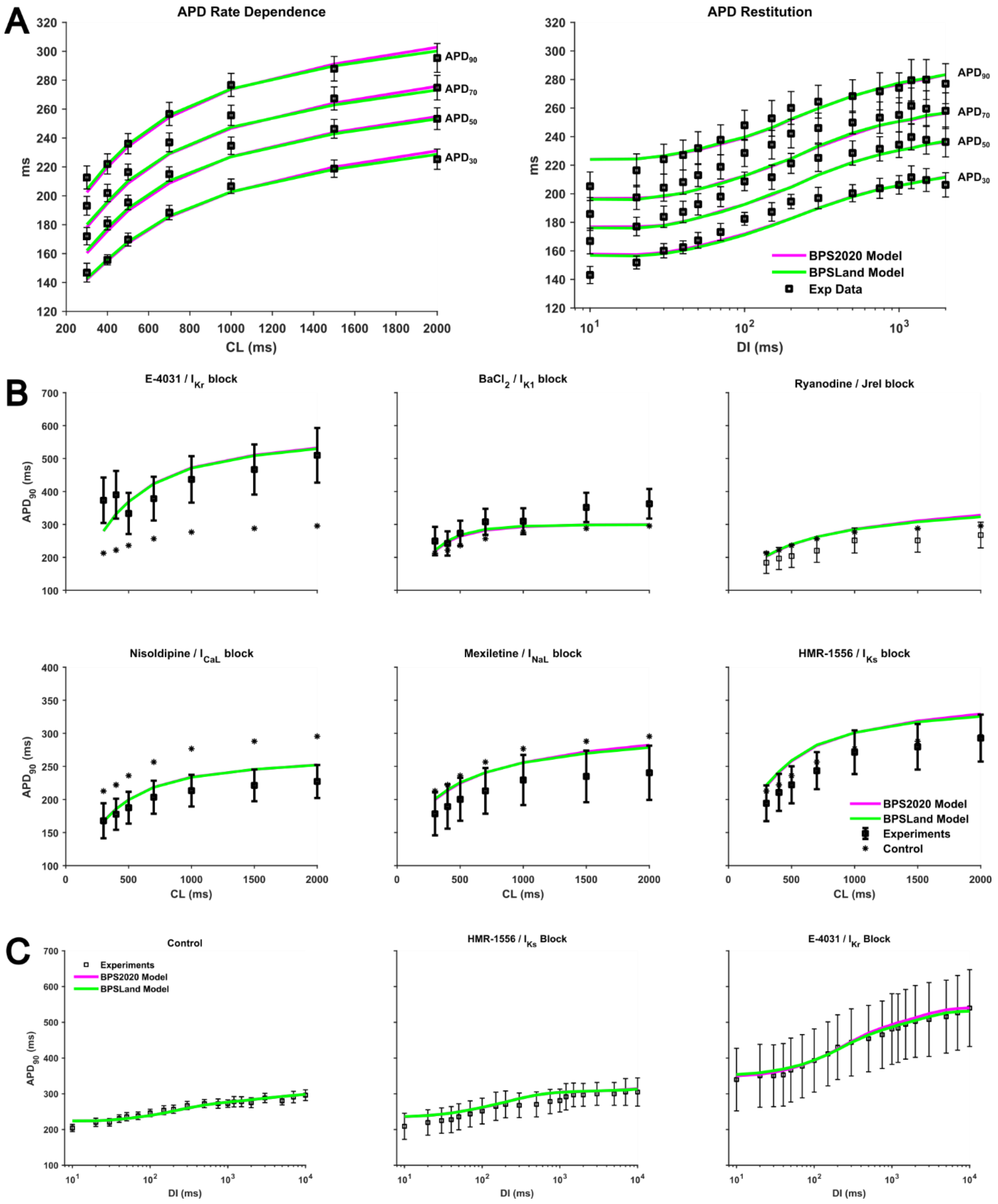

Figure S2. Rate dependence properties of BPS2020 vs. BPSLand. In all panels, simulation results for BPS2020 and BPSLand are shown in magenta and green, respectively. Experimental data from (O'Hara et al., 2011) are shown as black squares. **(A)** Steady state action potential duration (APD) rate dependence (CL – cycle length) and APD restitution obtained with the S1S2 protocol (DI – diastolic interval). APDs computed at 30, 50, 70, and 90% of repolarization are labeled on the right. **(B)** Steady

state APD_{90} rate dependence changes induced by specific current blocks; stars are the APD_{90} values in control conditions. **(C)** APD_{90} restitution changes induced by specific current blocks.

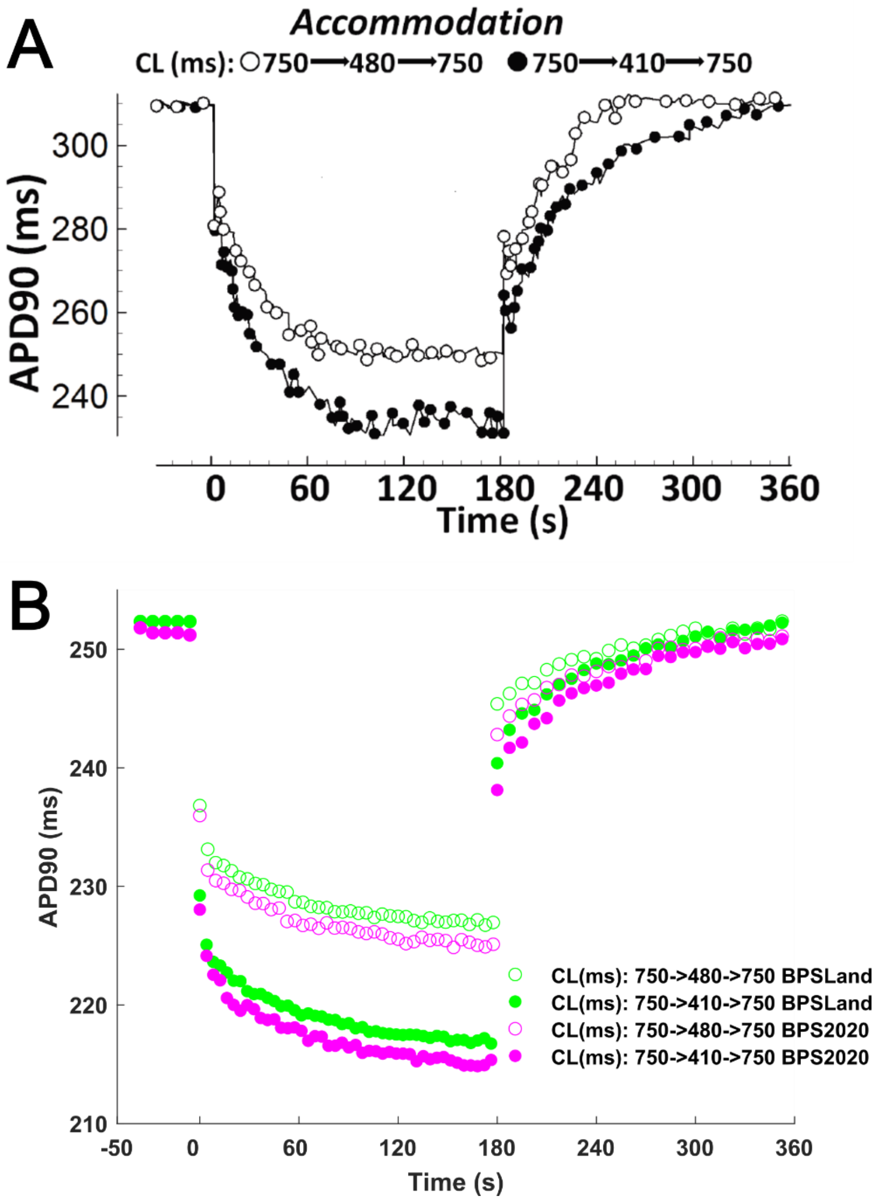

Figure S3. APD_{90} accommodation. At t = 0 s, the pacing cycle length (CL) is abruptly reduced from 750 to 480 ms (empty circles) or 410 ms (full circles). At t = 180 s, the CL is abruptly increased to its original value. **(A)** Action potential duration (APD) accommodation measured experimentally by (Franz et al., 1988). **(B)** APD accommodation simulated with the BPS2020 vs BPSLand model (magenta vs green traces, respectively). Panel **(A)** is adapted from (O'Hara et al., 2011).

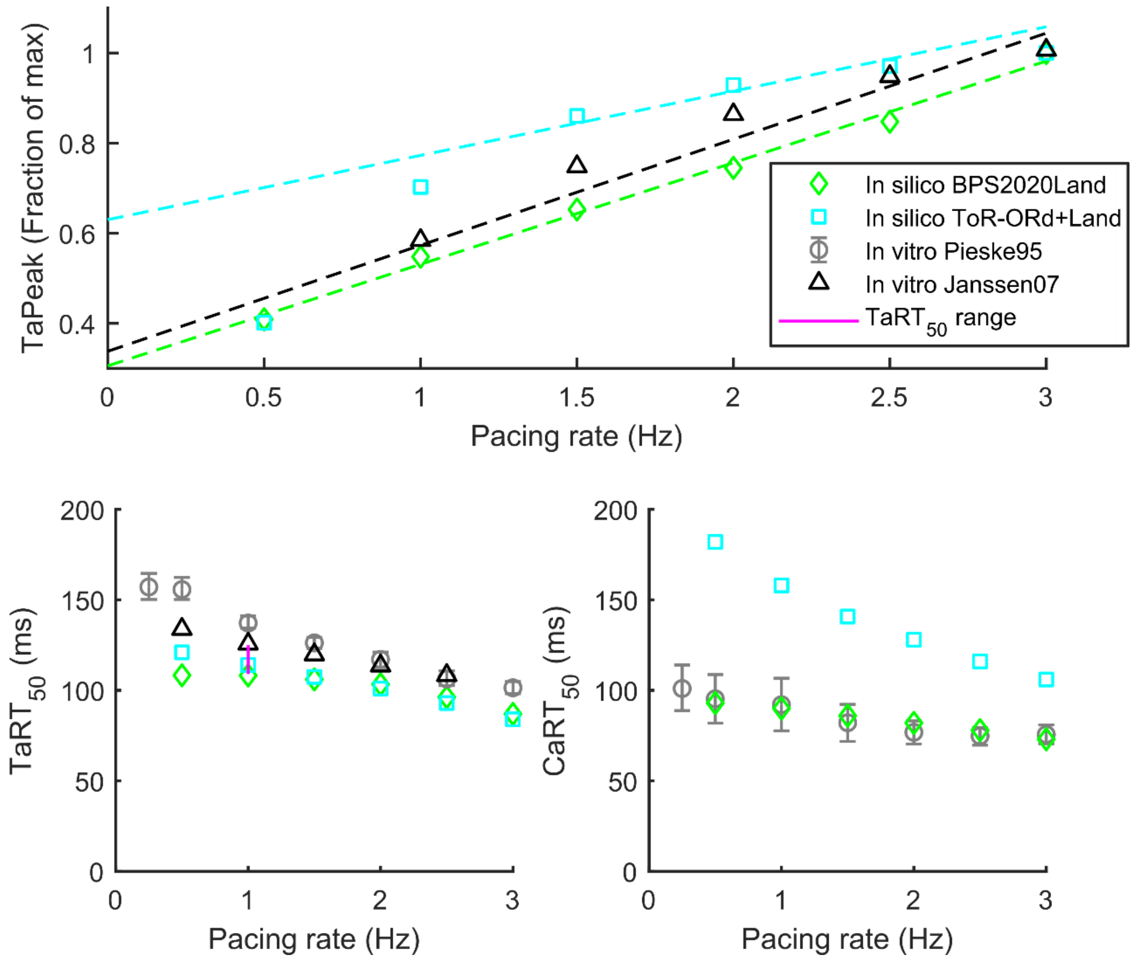

Figure S4: Active tension dependence on pacing rate and comparison with the in vitro data as in Figure 2 of the manuscript, simulated with BPSLand (green markers) and ToR-ORd+Land (cyan markers) models.

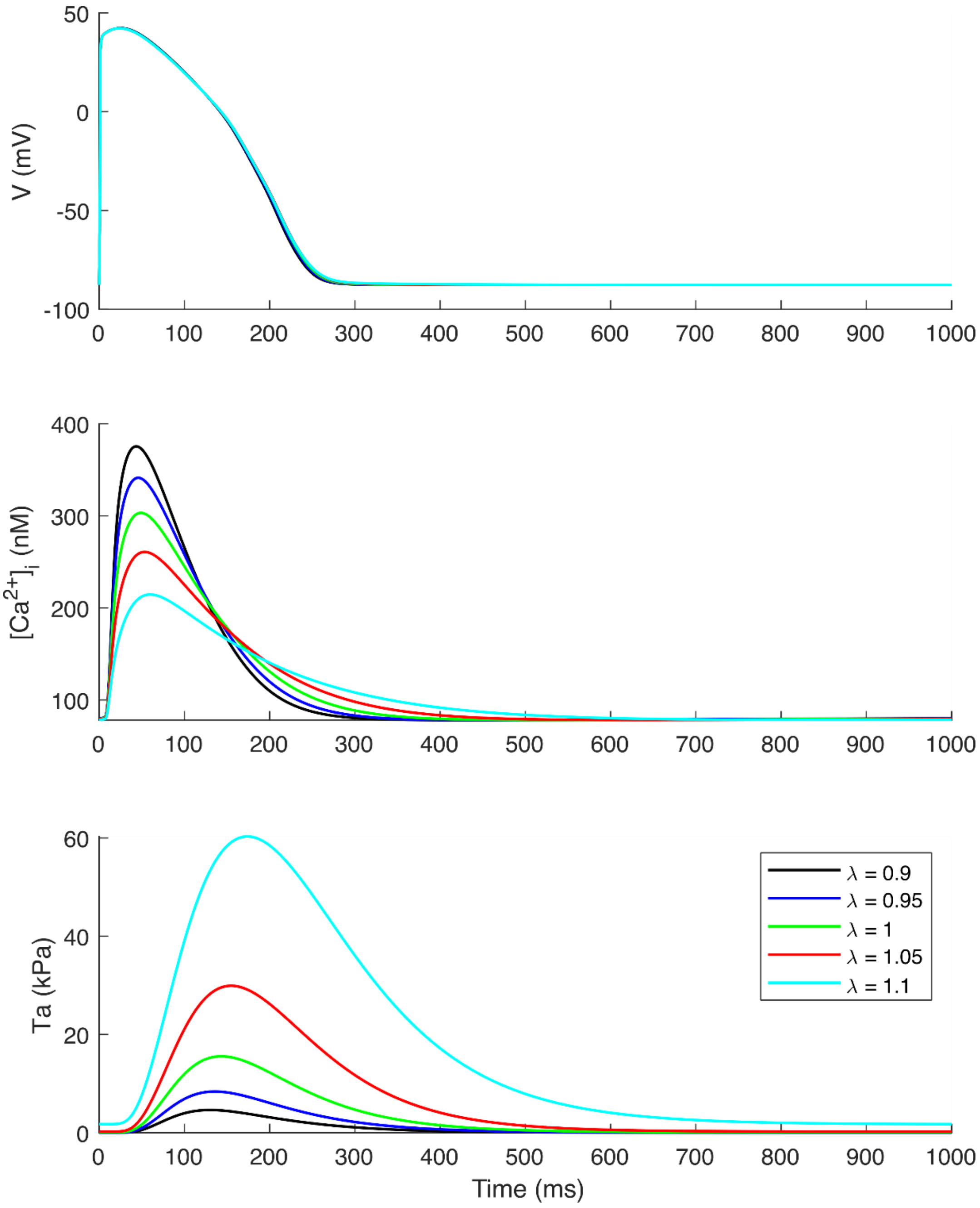

Figure S5. Length-dependence of action potential (top panel), calcium transient (middle panel), and active tension (bottom panel) simulated using the BPSLand model. The experimental results reported by (Vahl et al., 1997; Holubarsch et al., 1998) are qualitatively reproduced by the BPSLand model, although a quantitative comparison with experimental data is challenging due to the differences between the experimental and simulation setups.

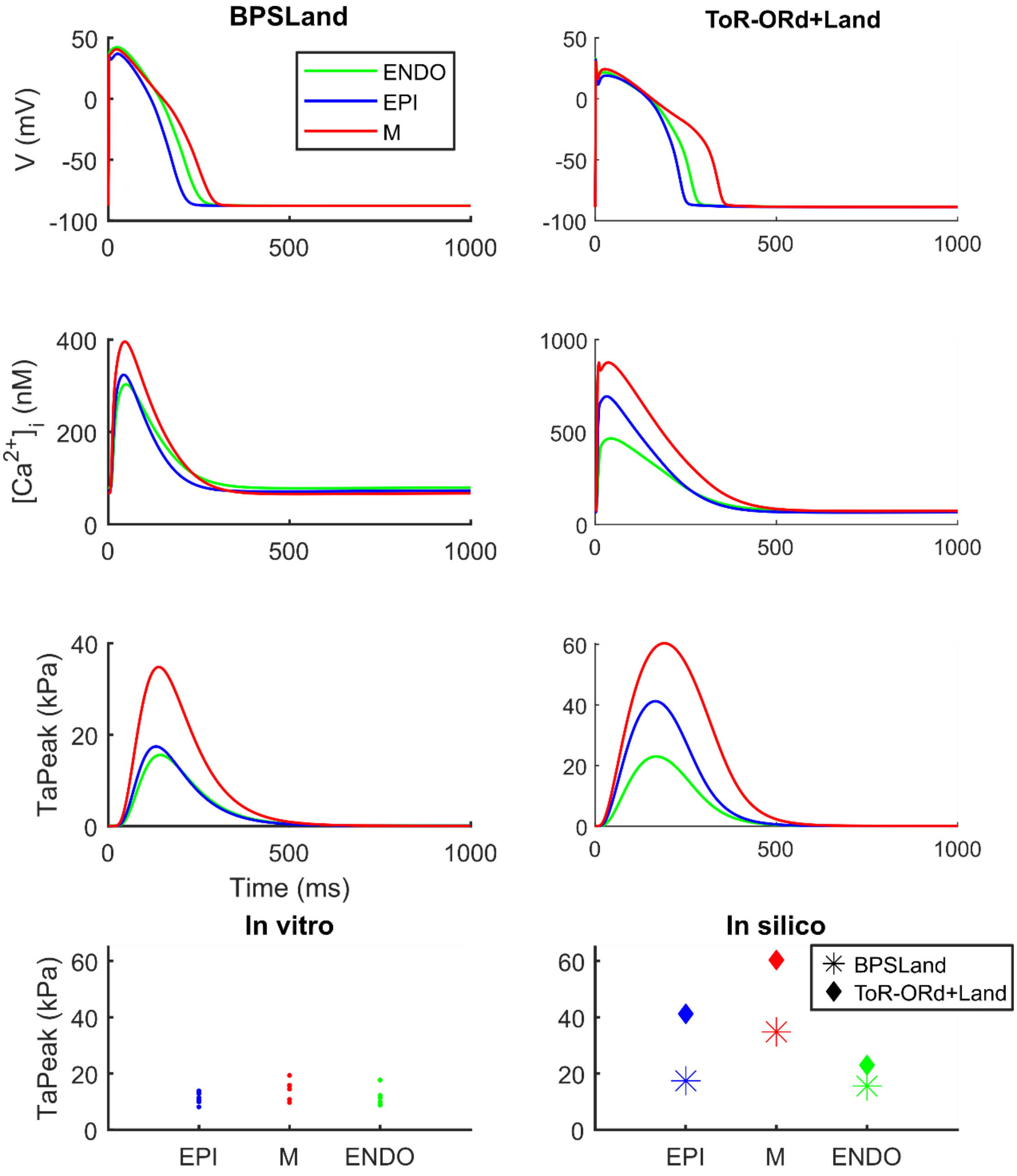

Figure S6: Comparison of the transmural heterogeneity simulations with BPSLand and ToR-ORd+Land models.

3 Supplementary Tables

Table S2: Weights (w_i) for each biomarker in the cost function.

Biomarker	w_i
TaPeak (kPa)	25
TaTTP (ms)	1
$TaRT_{95}$ (ms)	10
$TaRT_{50}$ (ms)	1
min(Ta) (kPa)	50

Table S3: Parameter changes during the optimization steps.

	BPS2020	LandCE	After Step 1	After Step 2	After Step 3 (BPSLand)
k_u (1/ms)	---	1	1.5230	1.5230	1.5230
ntm	---	5	3.0899	3.0899	3.0899
ν	---	7	1.0002	1.0002	1.0002
μ	---	3	2.0779	2.0079	2.0079
$[Ca^{2+}]_{T50}$ (μM)	---	0.8050	0.5000	0.5000	0.5000
$J_{rel,max}$ (1/ms)	0.0200	---	0.0200	0.0240	0.0220
$J_{up,max}$ (mM/ms)	3.130	---	3.130	3.1333	3.0000

Table S4: Action potential, Ca^{2+} transient and active tension biomarker changes during the optimization steps biomarker ($[K^+]_o$ = 5.4 mM, ENDO model).

	BPS2020	After Step 1	After Step 2	After Step 3 (BPSLand)
APD_{90} (ms)	239.9	239.6	234.7	239.9
APD_{50} (ms)	177.1	176.4	172.1	175.9
APD_{40} (ms)	160.1	159.4	154.1	158.9
Tri_{9040}	79.8	80.2	80.6	81.0
dV/dt_{max}	248.1	248.1	250.0	248.8
Vpeak	42.2	42.2	42.3	42.2
RMP	-87.6	-87.6	-87.7	-87.7
CTD_{90} (ms)	247.9	245.6	241.7	251.3
CTD_{50} (ms)	124.1	135.4	134.1	138.9
CaSys (mM)	316.3	299.2	308.7	303.3
CaAmp (mM)	235.1	218.5	235.3	225.0
CaDias (mM)	81.2	80.6	73.4	78.2
TaPeak (kPa)		14.6	16.1	15.6
TaTTP (ms)		142.4	141.1	142.9
$TaRT_{95}$ (ms)		306.2	304.6	307.4
$TaRT_{50}$ (ms)		107.2	105.6	108.4
TaMin (kPa)		0.110	0.084	0.100

$[Ca^{2+}]_o$ – APD_{90}	OK	OK	NO	OK

Table S5: APD_{90} values at different extracellular Ca^{2+} concentrations for BPSLand and for the intermediate model obtained before the manual tuning of the maximum J_{rel} and J_{up} fluxes (Column After Step 2 in Table S3).

APD_{90} (ms)	$[Ca^{2+}]_o$ (mM)		
	0.9	1.8	2.4
After Step 2	246.4	234.7	234.9
BPSLand	251.4	239.9	237.1

Table S6: Scaling factors for implementing the transmural heterogeneity.

	Epi/endo	M/endo
G_{NaL}	0.7	1
G_{to}	4	4
P_{Ca}, P_{CaNa}, P_{CaK}	1.4	2
G_{Kr}	1.1	0.8
G_{Ks}	1.4	1
G_{K1}	1.2	1.3
G_{NCXi},G_{NCXss}	1.2	1.4
G_{NaK}	0.9	0.7
G_{Kb}	0.6	1
$J_{rel,NP}$, $J_{rel,CaMK}$	1	1.7
$J_{up,NP}$, $J_{up,CaMK}$	1.3	1
$\overline{[CMDN]}$	1.2	1

Table S7: quinidine IC_{50} and Hill coefficients

	IC_{50}	Hill's coefficient
I_{NaF}	14.6	1.22
I_{Kr}	0.343	1
I_{CaL}	6.4	0.68
I_{Ks}	4.899	1.4
I_{to}	3.487	1.3

References

Bartolucci, C., Passini, E., Hyttinen, J., Paci, M., and Severi, S. (2020). Simulation of the Effects of Extracellular Calcium Changes Leads to a Novel Computational Model of Human Ventricular Action Potential With a Revised Calcium Handling. *Front. Physiol.* 11, 1–20. doi:10.3389/fphys.2020.00314.

Biliczki, P., Virág, L., Iost, N., Papp, J. G., and Varró, A. (2002). Interaction of different potassium channels in cardiac repolarization in dog ventricular preparations: Role of repolarization reserve. *Br. J. Pharmacol.* 137, 361–368. doi:10.1038/sj.bjp.0704881.

Dutta, S., Chang, K. C., Beattie, K. A., Sheng, J., Tran, P. N., Wu, W. W., et al. (2017). Optimization of an in silico cardiac cell model for proarrhythmia risk assessment. *Front. Physiol.* 8, 1–15. doi:10.3389/fphys.2017.00616.

Franz, M. R., Swerdlow, C. D., Liem, L. B., and Schaefer, J. (1988). Cycle length dependence of human action potential duration in vivo. Effects of single extrastimuli, sudden sustained rate acceleration and deceleration, and different steady-state frequencies. *J. Clin. Invest.* 82, 972–979. doi:10.1172/JCI113706.

Holubarsch, C., Lüdemann, J., Wiessner, S., Ruf, T., Schulte-Baukloh, H., Schmidt-Schweda, S., et al. (1998). Shortening versus isometric contractions in isolated human failing and non-failing left ventricular myocardium: Dependency of external work and force on muscle length, heart rate and inotropic stimulation. *Cardiovasc. Res.* 37, 46–57. doi:10.1016/S0008-6363(97)00215-0.

O'Hara, T., Virág, L., Varró, A., and Rudy, Y. (2011). Simulation of the Undiseased Human Cardiac Ventricular Action Potential: Model Formulation and Experimental Validation. *PLoS Comput. Biol.* 7, e1002061. doi:10.1371/journal.pcbi.1002061.

Thomas, N. L., and Williams, A. J. (2012). Pharmacology of ryanodine receptors and Ca2+-induced Ca2+ release. *Wiley Interdiscip. Rev. Membr. Transp. Signal.* 1, 383–397. doi:10.1002/wmts.34.

Tripathy, L. X. A., Pasek, D. A., and Meissner, G. (1998). Potential for Pharmacology of Ryanadine Receptor/Calcium Release Channelsa. *Ann. N. Y. Acad. Sci.* 853, 130–148. doi:10.1111/j.1749-6632.1998.tb08262.x.

Vahl, C. F., Timek, T., Bonz, A., Kochsiek, N., Fuchs, H., Schäffer, L., et al. (1997). Myocardial length-force relationship in end stage dilated cardiomyopathy and normal human myocardium: Analysis of intact and skinned left ventricular trabeculae obtained during 11 heart transplantations. *Basic Res. Cardiol.* 92, 261–270. doi:10.1007/BF00788521.

Walsh, K. B., Zhang, J., Fuseler, J. W., Hilliard, N., and Hockerman, G. H. (2007). Adenoviral-mediated expression of dihydropyridine-insensitive L-type calcium channels in cardiac ventricular myocytes and fibroblasts. *Eur. J. Pharmacol.* 565, 7–16. doi:10.1016/j.ejphar.2007.02.049.

Zucchi, R., and Ronca-Testoni, S. (1997). The sarcoplasmic reticulum Ca2+ channel/ryanodine receptor: Modulation by endogenous effectors, drugs and disease states. *Pharmacol. Rev.* 49, 1–

50.
